# Correction to “Bifurcated Hydrogen Bonds in a Peptide Crystal Unveiled by X-ray Diffraction and Polarized Raman Spectroscopy”

**DOI:** 10.1021/acs.cgd.3c00787

**Published:** 2023-07-27

**Authors:** Kazunori Motai, Nao Koishihara, Takuma Narimatsu, Hiroyoshi Ohtsu, Masaki Kawano, Yuki Wada, Kazuki Akisawa, Koji Okuwaki, Takehiko Mori, Ji-Seon Kim, Yuji Mochizuki, Yuhei Hayamizu

There are ten mistakes in the original paper that have been mentioned and are corrected below.

Page 4559: The caption of Figure 4 was incorrect, and the correct caption is

**Figure 4**. A) and B) Respective peak positions of Amide I and A band of FEFE peptide as a function of N···O distance. C) and D) Comparison of our data (red dots) with previous works (black dots) for the Raman shift of various molecular crystals with distinct hydrogen bonds. Data points of C and D refer to refs 21 and 22, respectively.

The citation number in the main text was wrong, and the corrected text is

To investigate the charge transfer of hydrogen bonding, CAFI (Configuration Analysis for Fragment Interaction)^26^ was utilized. This method allows for both quantitative and visual analysis of the charge transfer associated with hydrogen bonds in a directional manner. In the calculation, we selected three peptides in the unit cell obtained from the sc-XRD, which are aligned in the direction of the *a*-axis and *b*-axis. The directions and energetic contributions (in kcal/mol) of charge transfer are shown in Figure 5 (also Figure S13 and Tables S2 and S3). The illustrated results indicate the bifurcation of hydrogen bonds, where the contribution of the carboxy group at the side chain of glutamic acids was larger than that of the hydrogen bond formed by the Amide backbone. These results support the slow slope in Figure 4, where the interatomic distance of N···O along the *a*-axis has a relatively weak dependence on the frequency shift. The bifurcated hydrogen bonds should be considered in the spectroscopic characterizations of peptide crystals with a hydrogen bond network.

The bifurcated hydrogen bonds have essential effects on the structural stabilization and functionalization of proteins, such as the structural stabilization between layers of the multilayer β-sheet structure in silk fibroin^27^ and the supply of low-energy-barrier protons in enzyme catalysis.^28^ Amides with bifurcated hydrogen bonds show a large peak shift observed in IR and NMR spectroscopy.^25,27^ Combining our findings with previous knowledge about proteins provides an insight into the effects of bifurcated hydrogen bonds on the material design of peptide assemblies or crystals.

Page 4561: Corrected references are

(26) MochizukiY.A configuration analysis for fragment interaction.Chem. Phys. Lett.2005, 410 ( (4–6), ), 247–253. DOI: 10.1016/j.cplett.2005.05.079

(27) GomesS.; NumataK.; LeonorI. B.; ManoJ. F.; ReisR. L.; KaplanD. L.AFM Study of Morphology and Mechanical Properties of a Chimeric Spider Silk and Bone Sialoprotein Protein for Bone Regeneration.Biomacromolecules2011, 12 ( (5), ), 1675–1685. DOI: 10.1021/bm200060521370930PMC3090475

(28) NicholsD. A.; HargisJ. C.; SanishviliR.; JaishankarP.; DefreesK.; SmithE. W.; WangK. K.; PratiF.; RensloA. R.; WoodcockH. L.; ChenY.Ligand-Induced Proton Transfer and Low-Barrier Hydrogen Bond Revealed by X-Ray Crystallography.J. Am. Chem. Soc.2015, 137 ( (25), ), 8086–8095. DOI: 10.1021/jacs.5b0074926057252PMC4530788

(29) LiuA.; LuZ.; WangJ.; YaoL.; LiY.; YanH.NMR Detection of Bifurcated Hydrogen Bonds in Large Proteins.J. Am. Chem. Soc.2008, 130 ( (8), ), 2428–2429. DOI: 10.1021/ja710114r18247612

